# Root contact responses and the positive relationship between intraspecific diversity and ecosystem productivity

**DOI:** 10.1093/aobpla/plv053

**Published:** 2015-05-19

**Authors:** Lixue Yang, Ragan M. Callaway, Daniel Z. Atwater

**Affiliations:** 1 Division of Biological Sciences and the Institute on Ecosystems, The University of Montana, Missoula, MT 59812, USA; 2 School of Forestry, Northeast Forestry University, Harbin 150040, China; 3 Department of Plant Pathology, Physiology, and Weed Science, Virginia Tech University, Blacksburg, VA 24061, USA

**Keywords:** Ecosystem productivity, identity recognition, intraspecific genetic diversity, *Pseudoroegneria spicata*, root interactions

## Abstract

High species and functional group richness often has positive effects on ecosystem function including increasing productivity. Recently, intraspecific diversity has been found to have similar effects, but because traits vary far less within a species than among species we have a much poorer understanding of the mechanisms by which intraspecific diversity affects ecosystem function. We explored the potential for identity recognition among the roots of different *Pseudoroegneria spicata* accessions to contribute to previously demonstrated overyielding in plots with high intraspecific richness of this species relative to monocultures. First, we found that when plants from different populations were planted together in pots the total biomass yield was 30 % more than in pots with two plants from the same population. Second, we found that the elongation rates of roots of *Pseudoroegneria* plants decreased more after contact with roots from another plant from the same population than after contact with roots from a plant from a different population. These results suggest the possibility of some form of detection and avoidance mechanism among more closely related *Pseudoroegneria* plants. If decreased growth after contact results in reduced root overlap, and reduced root overlap corresponds with reduced growth and productivity, then variation in detection and avoidance among related and unrelated accessions may contribute to how ecotypic diversity in *Pseudoroegneria* increases productivity.

## Abstract (precis)

We show that root-root recognition processes may help to explain how genetic diversity within species can lead to great ecosystem functioning. Roots of plants from the same population slowed down much more after contacting each other than roots of plants from different populations, suggesting that mixing genetic diversity may lead to greater root overlap and thereby greater ecosystem functioning.

## Introduction

Biodiversity and ecosystem functioning are often positively linked (Balvanera *et al*. 2006; Cardinale *et al*. 2007, 2011). Among the most prominent explanations for why diversity improves ecosystem function is that different species complement or facilitate each other in chemical, spatial or temporal resource use (Eisenhauer 2012), and that more species-rich communities experience less suppression by pathogenic fungi than monocultures (Maron *et al*. 2011; Schnitzer *et al*. 2011; Kulmatiski *et al*. 2012). Furthermore, recent studies have shown that the root growth of some species increases in species mixtures compared with monocultures causing belowground overyielding, potentially due to reduced effects of plant pathogens in diverse assemblages (Mommer *et al*. 2010; de Kroon *et al*. 2012).

Most studies have focussed on the roles of species and functional group diversity on ecosystem functioning; however, a few recent studies have found that intraspecific diversity can have similar effects on ecosystem function (Crutsinger *et al*. 2006; Fridley and Grime 2010; Cook-Patton *et al*. 2011; Crawford and Rudgers 2012; Schöb *et al*. 2015). Similarly, Atwater and Callaway (2015) found that intraspecific diversity of *Pseudoroegneria spicata* (Pursh) Á. Löve increased productivity to a similar degree as generally reported in the literature for species diversity. This overyielding was primarily due to complementary interactions among accessions; however, there was no evidence that overyielding was related to resource depletion, a commonly cited mechanism for overyielding in species-diverse communities. Thus the mechanisms that lead to overyielding by diverse assemblages of *Pseudoroegneria* remain poorly understood.

A possible, but to our knowledge unexplored, mechanism for variation in productivity in ecotypic monocultures is different degrees of overlap among the roots of individual plants (see Schenk *et al*. 1999; Novoplansky 2009). Spatially segregated root systems have been documented among conspecifics at the scale of whole root systems and individual roots (Schenk *et al*. 1999), and segregation appears to be affected in some cases by the changes in root growth of one plant in response to contact with the roots of another plant, or identity recognition (Mahall and Callaway 1992; Cahill *et al*. 2010; Cahill and McNickle 2011). For example, root segregation may provide competitive advantages for resources or space for some individuals over others, functioning effectively as the establishment of territories (Schenk *et al*. 1999). It is unknown how spatial root segregation might affect the growth of individual plants, and how this ramifies to community productivity. Furthermore, root responses to other roots can be highly complex, including both decreased and increased growth rates after contact.

There is substantial evidence for different forms of identity recognition among the roots of individual plants, among individuals within populations, among populations and among species (Mahall and Callaway 1991, 1992; Krannitz and Caldwell 1995; Schenk *et al*. 1999; Gersani *et al*. 2001; Callaway 2002; Falik *et al*. 2003; Semchenko *et al*. 2007; Metlen *et al*. 2009; Novoplansky 2009; Bhatt *et al*. 2011). Mahall and Callaway (1996) explored self-non-self recognition among different populations of *Ambrosia dumosa* and found that the roots of plants demonstrated sharp declines in growth after contact with the roots of another plant from the same population. This decline did not occur when roots contacted the roots of an individual from a distant population. Others have reported various forms of identity recognition and subsequent change in root behaviour (de Kroon and Visser 2003; Donohue 2003; Cheplick and Kane 2004; Gruntman and Novoplansky 2004; Dudley and File 2007) and many of these studies have explored the potential for such recognition to affect interactions among and within species (Falik *et al*. 2006; see review by Novoplansky 2009). Recently, Semchenko *et al*. (2014) found that root exudates can communicate information about genetic relatedness, population origin and species identity. However, to our knowledge, no studies have explored how the responses of roots to other roots might contribute to how ecotypic diversity increases ecosystem function.

Because of this lack of research, and the wide range of ways that roots respond to each other and to resources, it is hard to predict whether contact and avoidance (i.e. territorial-like responses) or increased growth response to contact (e.g. Gersani *et al*. 2001; Novoplansky 2009; Fang *et al*. 2013) might increase overall productivity (see Milla *et al*. 2009). It is reasonable to predict that decreased root overlap might result in the improved growth of individuals in a community but not greater productivity overall. On the other hand, high root overlap has the potential to increase productivity via more complete root exploration of the soil and resource uptake (de Kroon and Visser 2003; de Kroon *et al*. 2012). Thus exploring correlations between root–root responses and productivity have the potential to resolve important general questions about the ecosystem ramifications of root behaviour.

Here we explored potential mechanisms for the positive relationship between ecotypic diversity of *P. spicata* and productivity reported by Atwater and Callaway (2015) in which plots with high intraspecific richness overyielded relative to monocultures. Specifically, we tested the hypotheses that (i) the total biomass of two interacting plants from different populations would be more than that for two plants from the same population and (ii) the growth rates of *Pseudoroegneria* roots would decrease more when contacting roots from other individuals from the same populations than when contacting roots from individuals from other populations.

## Methods

We used the same populations of *P. spicata* studied by Atwater and Callaway (2015). We obtained seeds of *Pseudoroegneria* from 12 sites throughout western North America, and with one exception, seeds were field collected in Montana or acquired from true-bred lines managed by the USDA Plant Germplasm Introduction and Testing Research Station in Pullman, Washington, USA. The one exception was a high-yielding wild-selected cultivar from south-eastern Washington, ‘Goldar’, which we purchased because of problems with seed viability of some of the naturally collected accessions.

To compare intra-genotype and inter-genotype interactions in control conditions, in May 2012 we sowed seeds from each of the 11 genotypes into 200 mL rocket pots to establish treatments in which plants were grown alone or with another individual. Six individuals from each population (accession) were grown alone (total *n* = 66), eight individuals were grown with another individual from the same population (total *n* = 88) and one individual from each population was grown with one other plant from each of the other populations (total *n* = 55). The pots were re-organized randomly on the bench once per week and watered every 2 days. Seedlings were harvested in August 2013, dried at 60 °C and total biomass was weighed. We analysed the effects of treatment on root mass and total biomass using linear mixed models with treatment (grown alone, with a plant from the same population or with a plant from another population) as a fixed effect and the identity of each interacting plant (i.e. its population) as random effects. We specified alone-vs-same population and alone-vs-other population as contrasts, and also calculated differences between biomass with same- and other-population as *post hoc* contrasts.

In a second experiment we monitored the growth rates of roots of individual *Pseudoroegneria* plants when they grew into the rhizospheres of other plants and made contact with roots of the same accession or different accession in the same chambers used and described in Mahall and Callaway (1991). These chambers were 20.5 × 12.5 × 2 cm, inside dimensions and were filled with 30 grit silica sand. Chambers were oriented at a 45° angle so that geotropic roots would grow down against Plexiglas viewing windows that could be covered and uncovered with shutters that excluded light. These chambers were placed in a greenhouse and the experiment ran from early February to late April. We initiated the experiment with 10 chambers containing intra-populations pairs and 14 chambers containing inter-population pairs but after mortality this replication was reduced to *n* = 8 and *n* = 12. Our inter-population pairs did not include all possible combinations, and these were chosen randomly with the condition that no pair combination was repeated. Two times during the experiment the chambers were saturated with a solution of 1.2 g L^−1^ of water-soluble fertilizer (15-2-20, The Scotts Company, Marysville, OH, USA). Sand in the chambers was kept continuously moist. After plants were established in ‘target’ and ‘test’ chambers, these chambers were connected so that roots of a test plant would grow into the rhizosphere and roots of a target plant. Elongation rates of all test-plant roots were visible and measurable through the Plexiglas along which they grew. Every 2 days the locations of root tips of the test plants were measured and elongation rates were measured as the distance travelled over those 2 days. Most roots of the test plants ultimately made contact with the roots of target plants and we calibrated our comparisons of growth rates of intra-population and inter-population pairs by the day they made contact. This calibration allowed us to compare growth rates prior to contact to growth rates after contact. We analysed root growth using linear mixed models with treatment (growth with a plant from the same population or from another population) and sampling date as fixed effects and focal plant population, target plant population as random effects. This design enabled us to treat daily measurements of repeated measures and account for effects of target and test plant (Faraway 2005). Because a different combination of target and test plants was used in each root chamber, the effects of chamber were redundant with effects of target and test plant and were not included in the final model. We used rate of root growth over 2 days from each time point as the dependent variable. All analyses were done using package ‘lme4’ in R version 3.1.2 (R Core Team 2012; Bates *et al*. 2014). Parameter significance was estimated using Satterthwaite approximation with package ‘lmerTest’ (Kunetsova *et al*. 2013).

## Results

**Figure 1.**
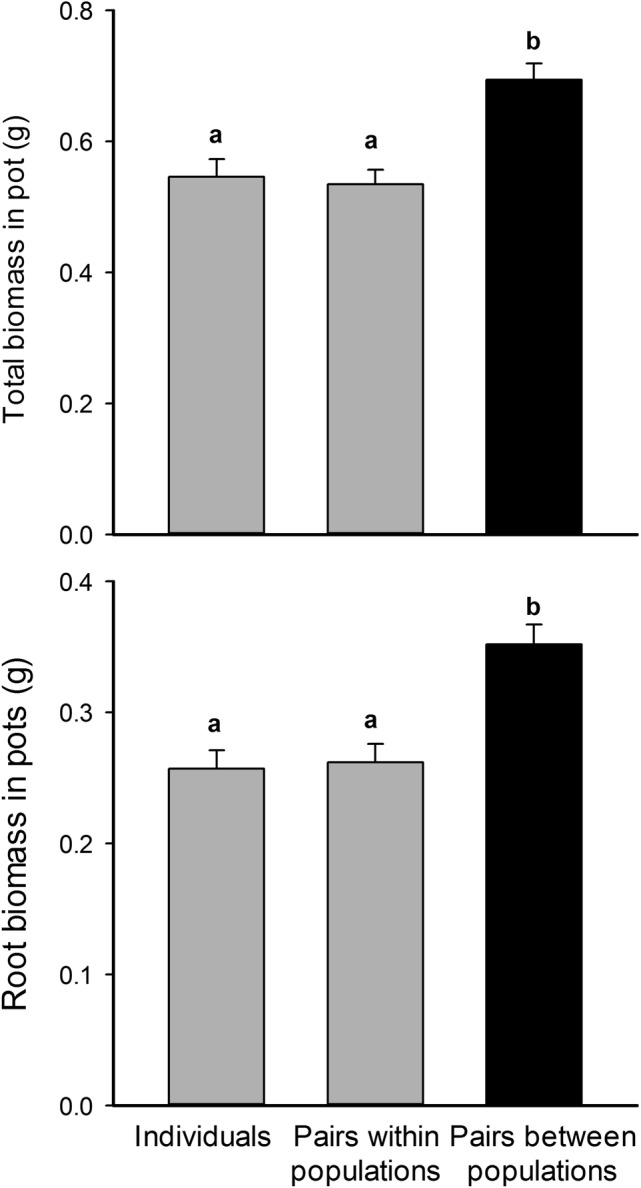
Total and root biomass in pots with either a *P. spicata* plant grown alone, grown with another plant of the same population or with a plant from a different population. Means that share a letter are not significantly different and error bars represent 1 SE.

When *Pseudoroegneria* individuals from the same population were planted together the total biomass yield in pots (both plants combined) was not greater than that of plants grown alone (Fig. 1). In other words, intra-population competition suppressed the growth of the two individuals to the point where total yield was the same regardless of the number of plants in per pot (*t*_same-vs-control_ = 0.014; df = 147.9; *P* = 0.989). In contrast, when plants from different populations were planted together the total biomass yield of pots was 30 % more than in pots with two plants from the same population (*post hoc*; *t*_same-vs-other_ = 4.238; df = 154.6; *P* < 0.0001) and 27 % more than in pots with individual *Pseudoroegneria* plants (*t*_other-vs-control_ = 4.133; df = 155.0; *P* < 0.0001). We found a similar but slightly stronger pattern for root biomass yield with a 37 % increase in pots with two plants from different populations relative to pots with two plants from the same population (*post hoc*; *t*_same-vs-other_ = 4.228; df = 154.5; *P* < 0.0001) and a 34 % increase relative to pots with one plant (*t*_other-vs-control_ = 4.566; df = 155.0; *P* < 0.0001). As for biomass yield, total root yield was not different when one plant was grown alone versus when two plants of the same population grew together (*t*_same-vs-control_ = 0.526; df = 148.3; *P* = 0.599). When we allowed a focal accession × treatment interaction in the model, this did not improve fit for total mass or root mass (log-likelihood comparison: *P* = 0.757, *P* = 0.999), and the random effects of focal accession were estimated to be very low (at least five orders of magnitude smaller than the residual variance). In sum, intra- and inter-population competition suppressed the growth of individual plants, but the total yield of the plants in inter-population combinations was less suppressed than it was in intraspecific combinations.

**Figure 2.**
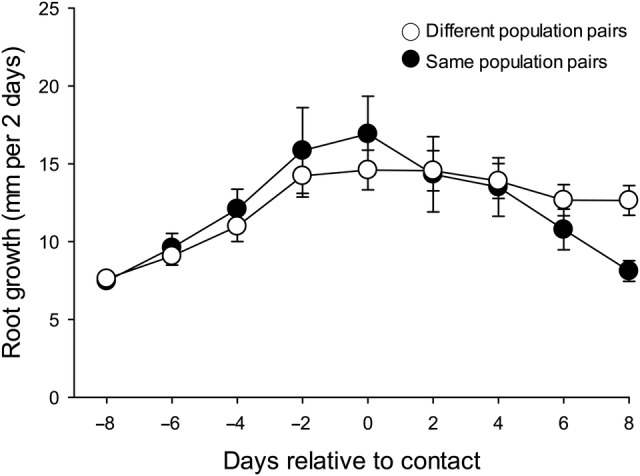
Rates of elongation of roots of *P. spicata* plants grown towards and making contact with roots of other *P. spicata* plants from either the same population or a different population. Elongation rates were standardized in time by aligning at Day 0 the days on which their contact with a target root was recorded (see Mahall and Callaway 1996). Error bars represent 1 SE.

In the root chamber experiment, root growth rates in both treatments increased steadily until root contact, at which point they either stabilized or significantly slowed, depending on whether the competitors were from the same or different populations (Fig. 2; Table 1). At the days of contact and for 2 days prior to contact, plants with same-population neighbours grew 20 % faster than plants with neighbours from different populations (*P* < 0.0043). After contact the roots of test plants growing with a target plant from the same population rapidly decreased in growth rate. Differences in growth rates between same-population and different-population target plants were statistically significant 6 and 8 days after contact (*P* < 0.0418). By 8 days after contact, test plants growing with neighbours from the same population were growing 36 % slower than the roots of test plants making contact with a plant from a different population (Fig. 2; Table 1).

**Table 1.** Results of linear mixed model of rate of root growth (mm per 2 days) against days since initial contact and treatment (grown with same population or with other population), with target and focal plant identity as random effects. A positive value of *B* for treatment effects means that plants with same-population neighbours grew faster than plants with other-population neighbours. Eight days prior to contact was used as the reference category because treatment effects had not yet begun to appear.

	*B*	df	*T*	*P*
Day − 6	1.230 ± 0.610	665.8	2.018	0.0440
Day − 4	2.896 ± 0.610	665.8	4.749	<0.0001
Day − 2	6.041 ± 0.610	665.8	9.909	<0.0001
Day of contact	6.630 ± 0.610	665.8	10.875	<0.0001
Day + 2	6.659 ± 0.610	665.8	10.921	<0.0001
Day + 4	5.954 ± 0.610	665.8	9.766	<0.0001
Day + 6	5.191 ± 0.610	665.8	8.515	<0.0001
Day + 8	4.837 ± 0.610	665.8	7.933	<0.0001
Treatment	−0.274 ± 0.716	328.0	−0.382	0.7025
Treatment × Day − 6	0.676 ± 0.952	665.8	0.710	0.4780
Treatment × Day − 4	1.682 ± 0.952	665.8	1.768	0.0776
Treatment × Day − 2	2.743 ± 0.952	665.8	2.882	0.0041
Treatment × Day 0	2.729 ± 0.952	665.8	2.867	0.0043
Treatment × Day + 2	0.023 ± 0.952	665.8	0.024	0.9811
Treatment × Day + 4	−0.129 ± 0.952	665.8	−0.136	0.8920
Treatment × Day + 6	−1.941 ± 0.952	665.8	−2.039	0.0418
Treatment × Day + 8	−4.462 ± 0.952	665.8	−4.688	<0.0001

## Discussion

We found that apparent relatedness (from the same population) of *Pseudoroegneria* plants affected root growth both before and after initial root contact. Initial root growth was faster in target plants growing with a conspecific competitor from the same population, compared with those competing with a member of a different population. Immediately after initial root contact this pattern reversed, with rates of root growth sharply falling off in plants interacting with a plant from the same population. This suggests the possibility of some form of contact detection and avoidance among *Pseudoroegneria* plants that depends to some degree on relatedness. In a similar study, Krannitz and Caldwell (1995) measured elongation rates of *Pseudoroegneria* roots as they encountered conspecific roots, roots of the closely related *Agropyron desertorum* (*Pseudoroegneria* used to be classified as *Agropyron*) or roots of the unrelated *Artemisia tridentata*. Unlike our results, they did not find decreased growth after contact for conspecific interactions, but they did find that *Pseudoroegneria* roots sharply decreased elongation after contact with *Agropyron* roots and that this effect differed among *Pseudoroegneria* genotypes. *Agropyron* roots were not affected by *Pseudoroegneria* roots. These results were corroborated by Huber-Sannwald *et al*. (1996), who found that *Pseudoroegneria* appeared to recognize and respond differently to competition with conspecifics versus competition with *Agropyron*.

Root interactions have been widely discussed as potential drivers of the relationship between plant *species* diversity and ecosystem functioning (Wilson and Tilman 2002; Cahill 2003; Rajaniemi 2003; Rajaniemi *et al*. 2003; Mommer *et al*. 2010; de Kroon *et al*. 2012). But there are two conflicting ideas for how root interactions might function in this context. By far the most cited mechanism is that of niche partitioning; the idea that different species more fully occupy belowground niche space, more completely utilizing resources and thus increasing productivity. However, Mommer *et al*. (2010) and de Kroon *et al*. (2012) provided experimental evidence for an alternative mechanism. They found that the roots of grassland species overyielded in the presence of the roots of other species, substantially increasing root density without any evidence for spatial partitioning. Furthermore, this overyielding was connected to more beneficial soil biota in diversity species mixtures, linking their results to a body of other recent work demonstrating how soil biota have powerful overall effects on the species diversity–productivity relationship (Maron *et al*. 2011; Schnitzer *et al*. 2011). Our results, but for intraspecific diversity, are broadly supportive of the findings reported by de Kroon *et al*. (2012), in that we found that greater root overlap and root production among different accessions of *Pseudoroegneria* correlated with greater productivity in the field (Atwater and Callaway 2015) and in pots by mixes of accessions (Fig. 1). Put another way, the decrease in root growth after contact and the lower root growth demonstrated in intra-accession interactions corresponded with lower productivity in low accession-diversity plots in the field (Atwater and Callaway 2015). However, intraspecific relatedness in general can correspond with a wide range of ecosystem function. For example, Biernaskie (2011) found that differences in yield among mixes of identical genotypes of *Ipomea hederacea* was greater than that of mixed genotypes, suggesting that productivity might be higher for related individuals. However, to our knowledge, our study is the first to connect root–root interactions to intraspecific diversity–ecosystem function relationships. If increased root overlap increases the overall exploration of soil space and resource acquisition, then our results suggest that mixtures of population-accession might increase resource acquisition and growth due to increased root overlap.

In a similar, but interspecific example, Padilla *et al*. (2013) found that monocultures of *Festuca rubra* had higher root densities and a faster rate of soil nitrate depletion than monocultures of *Plantago lanceolata*, which indicated that the former was a superior competitor for nutrients. However, in experiments they found that *Festuca* was an inferior competitor to *Plantago*. In these experiments *Plantago* overyielded in root growth. They argued that that competitive superiority occurred through root growth stimulation by a competitor prior to nutrient depletion instead of superior ability to deplete nutrients. Inhibition after contact may also function as detection and avoidance mechanisms that might reduce competition among established and closely related plants, a form of territoriality (Schenk *et al*. 1999). This may explain the initially rapid root proliferation of *Pseudoroegneria* growing with neighbours from the same population. If more closely related neighbours are stronger competitors (Cahill *et al*. 2008), and if this pattern holds within species as well as among species (as in Cheplick and Kane 2004), increased root proliferation in the presence of members of the same population could be important for establishing competitive superiority. In our study, rapid initial root proliferation could be a form of territorial expansion. However, a dramatic reduction in proliferation following root contact suggests that there is a cost to further proliferation into territory already occupied by a related competitor. It is not immediately clear why this should be the case, or why we observed a different pattern in plants growing with a less related neighbour.

It is important to note that we used accessions of *Pseudoroegneria* (or potentially ecotypes) from across a very large portion of the regional distribution of the species. The seeds of each accession were pooled, and not single seed descent families. Thus we do not yet know whether enough genetic variation exists within populations to create important effects on overyielding in natural populations. However, by using such a wide breadth of *Pseudoroegneria* accessions we have explored the *potential* of intraspecific diversity–ecosystem function relationships and provided a working hypothesis for a mechanism.

Our results add to the growing body of evidence for how roots may respond to contact with other roots individual plants, among individuals within populations, among populations and among species (Mahall and Callaway 1991, 1992; Krannitz and Caldwell 1995; Schenk *et al*. 1999; Gersani *et al*. 2001; Callaway 2002; Falik *et al*. 2003; Semchenko *et al*. 2007; Metlen *et al*. 2009; Novoplansky 2009). However, the mechanisms by which closely related species might respond to each other are not known. Badri *et al*. (2012) found that more defence and stress-related proteins were released from roots when a specific control genotype of *Arabidopsis* was grown alone than when it was co-cultured with another homozygous individual or with an unrelated plant. They pointed out that their results suggested that plants can detect and respond to ecotypic variation in neighbours. In contrast, Gruntman and Novoplansky (2004) reported that self-/non-self-discrimination among *Buchloe dactyloides* plants was mediated by physiological coordination among roots developing on the same plant (also see Mahall and Callaway 1996). Furthermore, we do not know whether differences in root growth between different accessions are related to between-kin aspects of altruism or simply increased competition between non-related individuals.

In summary, our results link a growing body of literature on the capacity of roots from different species and different accessions to detect and respond to each other to how diverse mixtures of species or accessions enhance ecosystem function. We do not know whether similar mechanisms might also operate in species-diverse systems, but it warrants investigation.

## Sources of Funding

R.M.C. thanks the Montana Institute on Ecosystems and NSF EPSCoR Track-1 EPS-1101342 (INSTEP 3) for support.

## Contributions by the Authors

D.Z.A. developed the conceptual background of intraspecific diversity and ecosystem functions, R.M.C. developed the conceptual background of root–root interactions. R.M.C. and L.Y. designed the experiment, L.Y. carried out the experiment and all authors wrote the paper.

## Conflict of Interest Statement

None declared.
